# Feedback as a two-way process: how relational dynamics shape learning in workplace-based training

**DOI:** 10.1186/s12909-026-09462-7

**Published:** 2026-05-18

**Authors:** Dania Alkhiyami, Muhammad Abdul Hadi, Ahsan Sethi, Tarik Al-Diery

**Affiliations:** 1 QU Health, Qatar University, Doha, Qatar; 2 Hamad Medical Corporation, Doha, Qatar; 3 College of Pharmacy, QU Health, Qatar University, Doha, Qatar; 4 Health Professions Education, Department of Public Health, College of Health Sciences, QU Health, Qatar University, Doha, Qatar

**Keywords:** Feedback, Workplace-based learning, Metacognition, Experiential learning, Psychological safety

## Abstract

**Background:**

Providing constructive feedback is essential for supporting the growth and development of trainees during experiential learning. However, both preceptors and students often face challenges in effectively giving and receiving feedback, which can hinder learning and limit trainee progress. This study explores how relational dynamics between preceptors and trainees shape the interpretation, uptake, and developmental impact of feedback within workplace-based pharmacy training.

**Methods:**

An exploratory interpretivist qualitative design was used, drawing on semi-structured interviews conducted face-to-face or virtually between July 2023 and January 2024. A purposive sample of pharmacy students and preceptors were recruited from the community and hospital setting across Qatar. Data were transcribed verbatim and analyzed using reflexive thematic analysis to generate an in-depth understanding of participants’ experiences.

**Results:**

A total of 12 pharmacy students and 12 pharmacy preceptors participated in the study. Four interrelated themes were identified. Feedback was experienced as a relational practice shaped by trust, preceptor identity, and the quality of the preceptory relationship. Participants described feedback as a developmental catalyst that supported insight, confidence, and professional growth when it was timely, specific, and grounded in psychological safety. However, structural and relational tensions, including time pressures, variability in preceptor approaches, and discomfort with critical dialogue, often limited the depth and consistency of feedback. Finally, participants highlighted the need to build a shared feedback culture through clearer expectations, ongoing preceptor training, and systems that support reflection and co-regulated learning.

**Conclusion:**

Effective feedback was shaped not only by its timeliness and specificity but also by the quality of the preceptor-student relationship and the level of psychological safety within the learning environment. These findings suggest that feedback in experiential learning should be approached as a relational and bidirectional process that supports trainee insight, reflection, and progression toward autonomous practice.

**Supplementary Information:**

The online version contains supplementary material available at 10.1186/s12909-026-09462-7.

## Introduction

The focus on workplace-based training to prepare future autonomous pharmacists capable of navigating the complexities of an evolving profession requires a structurally sound system that can nurture and support trainee development [1, 2]. Experiential learning plays a central role in preparing trainees for autonomous practice by enabling them to consolidate knowledge, apply clinical reasoning, and develop professional identity within authentic workplace settings [3, 4]. Within these environments, feedback is widely recognized as a critical driver of learning and the nurturing of trainees in their professional journeys [5–7]. Constructive, timely, and learner-centered feedback supports growth in competence, reflection, and professional judgement in trainees [8]. Yet, decades of research across the health professions reveal that feedback often fails to achieve its developmental intent [9–11].

A persistent tension challenges the feedback process. Preceptors commonly struggle with delivering meaningful, constructive critique, while trainees grapple with receiving and applying feedback in ways that foster improvement [9, 12]. These difficulties stem from more than technical skill; they reflect underlying developmental and relational dynamics that are at times overlooked or poorly understood [13, 14]. Learners often have limited insight into their own performance, experience strong affective reactions to corrective feedback, and demonstrate underdeveloped metacognitive skills; factors that collectively hinder uptake and reflection [9, 15, 16]. Importantly, feedback is experienced as a relational process between supervisor and trainee, where hands-on coaching, mentorship, and sustained investment in trainee development shape how learners engage with and act upon feedback [13, 17, 18].

Feedback in workplace-based training does not occur in isolation; it is deeply embedded within assessment cultures that shape how feedback is interpreted [8, 19]. Research consistently shows that learners frequently perceive feedback – regardless of intent – as judgement rather than coaching [19]. When feedback “feels like assessment,” its developmental value diminishes, engagement decreases, and psychological safety erodes [19–21]. This dynamic is further complicated by the uneasy alliance between assessment and feedback, where preceptors are simultaneously expected to support learning and evaluate competence [19] Schut and colleagues articulated that learners’ perceptions of assessment and feedback are strongly shaped by the teacher-learner relationship [17] Teachers’ positional authority, expertise, and relational warmth significantly influence whether assessments are perceived as learning opportunities or as high-stakes judgements by learners [17, 22] However, little is known about how these relational dynamics unfold within experiential pharmacy education contexts.

These sociocultural dynamics reveal that effective feedback is not merely a matter of delivering information; it is a relational process shaped by trust, credibility, and clarity of purpose [14, 17, 22–24]. Both preceptors and trainees must have a shared understanding of the developmental intent of feedback, yet such clarity is often lacking within clinical training environments [14]. As a result, preceptors may default to overly positive or neutral feedback to protect student confidence, while trainees may prioritize maintaining face or avoiding perceived high-stakes judgement [9, 25]. These patterns reflect broader cultural influences and highlight a pressing need to better understand how feedback is experienced within real-world experiential learning contexts [26].

Although feedback is widely explored within higher education, it remains inconsistently internalized in pharmacy and health professions education [27–29]. Existing literature often emphasizes techniques or delivery mechanisms, with far less attention to the relational and contextual factors that fundamentally influence how feedback is interpreted, internalized, and acted upon [10, 30] As workplace-based learning becomes increasingly central to professional training [31], a deeper understanding of these interwoven relational dynamics is crucial to cultivating environments that nurture trainee growth, reflective capacity, and autonomy. Therefore, this study explores how relational dynamics between preceptors and trainees shape the meaning, reception, and developmental impact of feedback within workplace-based pharmacy training.

## Methods

### Study design

This study adopted an interpretivist qualitative design, using semi-structured interviews and reflexive thematic analysis to explore pharmacy students’ and preceptors’ experiences with feedback during experiential learning [32]. An interpretivist paradigm was selected to examine how participants engage with feedback and how relational and contextual processes shape these interactions within workplace-based learning [33]. Interviews were conducted in the State of Qatar between July 2023 and January 2024, either face-to-face or virtually, depending on participant preference. Ethical approval was obtained from the Hamad Medical Corporation Medical Research Center (MRC-01-23-426) and the Qatar University Institutional Review Board (1987201-1).

Semi-structured interviews were chosen as they enable participants to narrate their experiences in their own words, providing depth, nuance, and contextual richness to their lived experiences with feedback in experiential learning [34]. The interview guide was developed iteratively, beginning with the study objectives and informed by a review of the literature on feedback in experiential education [35, 36]. Content validation with subject-matter experts further ensured relevance, and a pilot interview led to additional refinements. Probing questions were incorporated throughout to support deeper exploration of emerging ideas and themes in the interview process. The full interview guide used for both students and pharmacy preceptors is provided in Appendix A.

### Study setting and participants

Participants were recruited from the Qatar University College of Pharmacy and its affiliated experiential training sites, including community pharmacies and Hamad Medical Corporation hospital settings in Doha, Qatar. As the College of Pharmacy is Qatar’s only pharmacy program overseeing undergraduate and workplace-based training, preceptors are formally affiliated with the program and participate in regular training on precepting and student development. Pharmacy students are introduced early in their training to metacognitive skill development and the application of feedback in practice [37]. Recruiting participants with prior exposure to reflective and feedback practices enabled the generation of rich accounts that illuminate the relational dynamics through which feedback is experienced and enacted.

Recruitment was conducted via email invitations containing detailed study information and consent to participate forms, with participation being voluntary. A purposive maximum variation sampling strategy was employed to capture diverse perspectives across training stages and practice settings. For students, we sought variation in academic year (years 3–5 and PharmD), number of completed rotations, and practice exposure (hospital and community). For preceptors, we aimed to recruit participants across different practice settings (community and hospital), years of precepting experience, and professional roles (staff pharmacist, manager, clinical specialist). As students and preceptors were known to the authors, participants were purposively identified and invited based on their demonstrated engagement with reflective learning (students) and extensive precepting experience and feedback practices (preceptors). All eligible participants who responded to the recruitment email and provided consent were interviewed.

The pharmacy profession and student body in Qatar are predominantly female, with admission of male students at Qatar University College of Pharmacy beginning only in 2021 [38]. Accordingly, the majority of recruited participants were female. Gender was not used as a sampling criterion, and no efforts were made to achieve a specific gender distribution.

### Data collection

One-on-one interviews were scheduled for sixty minutes and were audio-recorded with participant permission beforehand. Interviews were conducted either face-to-face or virtually, via Microsoft Teams (Microsoft Corporation, Redmond, Washington, USA), between July 2023 and January 2024. The lead investigator (DA) – who is also a clinical pharmacist and preceptor in a major metropolitan hospital – conducted all interviews. Throughout the interview process, the lead investigator utilized a semi-structured approach to elucidate the experiences of students and preceptors regarding the relational dynamics of how feedback is internalized, operationalized, and enacted. Interviews were conducted iteratively, with emerging insights discussed by the research team throughout data collection. These discussions informed subsequent interviews and guided ongoing recruitment to ensure variation across participant characteristics and depth of exploration of developing themes. No predetermined sample size was established. Recruitment of participants continued until sufficient information power was achieved and adequate variation across participant characteristics had been obtained, such that additional interviews did not yield substantively new insights relevant to the study aim [39]. Recruitment occurred iteratively and concurrently with data analysis, allowing the research team to monitor variation across participant characteristics and emerging patterns in the data.

Audio-recorded interviews were transcribed verbatim by DA to Microsoft Word (Microsoft Corporation, Redmond, Washington, USA). To maintain confidentiality, transcripts were anonymized using unique identifiers distinguishing preceptors (P) from students (S). Only de-identified transcripts were shared within the research team to facilitate analytic triangulation. Following each individual interview, the research team met to review field notes, reflect on key issues raised by participants, and discuss emerging patterns, areas of convergence, and points requiring further exploration. These discussions informed subsequent interviews and ongoing recruitment decisions.

Given the lead investigator’s positionality and prior professional relationships with some preceptors and students, reflexive notes were maintained throughout data collection to document initial impressions, contextual observations, and evolving analytic reflections. These notes supported the interpretive process by making explicit the researcher’s assumptions, professional experiences, and potential influences on data generation and analysis. Other members of the research team (MA and TD) also maintained reflexive journals throughout the data analysis process, acknowledging their roles as clinical educators and considering how their professional experiences may have shaped interpretation.

### Data analysis

This study was informed by an interpretivist paradigm, recognizing that participants’ experiences of feedback are socially constructed and contextually situated [32]. An inductive reflexive thematic analysis approach, guided by Braun and Clarke’s six-phase framework, was used to analyze the data [40]. Analysis was inductive and iterative, with the primary investigator (DA) and co-investigator (MA) engaging in repeated readings of the transcripts to develop familiarity with the dataset. Coding was conducted reflexively using Microsoft Word (Microsoft Corporation, Redmond, Washington, USA), with the primary investigator (DA) and co-investigator (MA) generating initial codes through iterative engagement with the data, capturing both descriptive meanings and interpretive insights. Codes were then compared, expanded, and consolidated into broader patterns of meaning. Following initial coding, the research team met across multiple sessions to collaboratively review and refine the coding framework. Through these discussions, interpretations of the data were critically examined and codes were iteratively refined.

Preliminary themes were generated through an iterative process of clustering and refining related codes. The research team engaged in ongoing dialogue to review generated themes, explore alternative interpretations, and ensure that analytic decisions were grounded in the data, thereby supporting team-based analytic triangulation. Themes were refined until they offered a coherent, credible account of participant experiences. An audit trail of analytic decisions and reflexive notes was maintained to enhance dependability and confirmability [41]. To enhance transparency and methodological rigor, data collection, analysis, and reporting were guided by the COREQ checklist [42], detailed in Appendix B.

The anonymized interview transcripts that support the findings of this study are not publicly available due to ethical and institutional restrictions. However, the data may be made available upon reasonable request, subject to approval by the Hamad Medical Corporation Medical Research Center (MRC-01-23-426) and the Qatar University Institutional Review Board (1987201-1).

## Results

A total of 24 participants consented to participate in one-on-one interviews, including 12 pharmacy students from four academic years and 12 pharmacy preceptors representing diverse practice settings and experience levels. An overview of participant characteristics is presented in Table 1.

**Table 1:** Characteristics of participants

Students		Preceptors	
Characteristics	Number (%)	Characteristics	Number (%)
Gender (Female)	12 (100)	Gender	
		Female	6 (50)
		Male	6 (50)
Academic year		Years of precepting	
Year 3	3 (25)	3–5	3 (25)
Year 4	1 (9)	6–10	6 (50)
Year 5	4 (33)	11–15	3 (25)
Doctor of pharmacy	4 (33)		
Number of rotations completed		Area of practice	
1	3 (25)	Community	4 (33)
2	1 (9)	Hospital	8 (67)
6	4 (33)	Position	
14	4 (33)	Pharmacist	5 (42)
		Pharmacy manager	1 (8)
		Clinical pharmacist	4 (33)
		Clinical pharmacist specialist	2 (17)

We identified four overarching themes that captured how feedback was understood and enacted within experiential learning: (1) feedback as a relational practice shaped by trust and preceptor identity, (2) feedback as a developmental catalyst that supports learner insight and progression toward autonomy, (3) structural and cultural tensions that constrain meaningful feedback, and (4) the need to build a shared feedback culture through clearer expectations, co-regulation, and enhanced preceptor support.

### Feedback as a relational practice shaped by trust and preceptor identity

Participants described feedback as more than the exchange of comments between a preceptor and a trainee. Instead, feedback was portrayed as a relational process in which trust, psychological safety, and mutual understanding strongly shaped how feedback was delivered, interpreted, and acted upon. Preceptors emphasized that their role extended beyond teaching; they saw themselves as role models responsible for nurturing trainees’ growth by establishing rapport and creating a safe, accountable environment for learning. As one preceptor expressed:

*“I think my role is not just being an educator*,* but it’s trying to be a role model for the future generation of pharmacists. My actions carry a lot of weight in how others see me”* (Int22P10).

Students echoed this view, explaining that preceptors who led by example were more likely to be trusted and perceived as credible sources of constructive feedback. They valued preceptors who demonstrated the professionalism, integrity, and communication standards expected in practice and viewed these behaviors as benchmarks for their own development:

*“It’s important that we see our preceptor as a role model*,* as we are the future generation of pharmacists. Seeing how they are will eventually influence how we become.”* (Int7S7).

Participants further emphasized that trust was strengthened through consistent guidance, mentorship, and the preceptor’s willingness to invest in the trainee’s progress. Students noted that hands-on coaching and supportive interactions fostered psychological safety, making them more open to receiving critical feedback and more confident in applying it. These relational dynamics were perceived as essential foundations for meaningful learning and for shaping emerging professional identity, as articulated by one student:

*“At the beginning of the rotation*,* I was very nervous because I knew she was evaluating me. I didn’t want to say something wrong. But after she started asking for my opinion and explaining why she corrected me and involving me in the discussions…I felt more comfortable. I realized she wasn’t trying to criticize me but push me to improve.”* (Int5S5).

### Feedback as a developmental catalyst supporting trainee insight, reflection, and progression toward autonomy

Participants viewed feedback as a central mechanism for trainee growth, emphasizing that constructive comments should be tailored, specific, and accompanied by clear, actionable plans revisited throughout the rotation. Both students and preceptors highlighted the need for feedback to occur in a supportive and private environment, where discussions could focus on progress, challenges, and goals without fear of embarrassment:

*“The environment really matters when giving feedback. It needs to be in a private*,* face-to-face setting. It shouldn’t feel like blaming the student*,* but more like an opportunity for them to reflect on their performance*,* think about their challenges*,* and plan how they can improve.”* (Int152P3).

Preceptors identified feedback as a driver of self-reflection and metacognitive development. When feedback was specific and individualized, students reported greater ability to recognize their strengths and areas for improvement, supporting deeper insight and self-regulated learning. However, preceptors described a tension: while they regarded reflection as essential for continuous development, some students appeared unaware of its importance or were unsure how to engage in it meaningfully. In some instances, preceptors perceived that limited learner insight contributed to dismissive responses to feedback, highlighting the central role of self-awareness and attitude in shaping receptiveness. As one preceptor explained:

*“One of the key aspects of assessment is the learner’s attitude. We often talk about knowledge and skills*,* but attitude is the driving force. If someone has a positive attitude*,* they’re more likely to benefit from feedback because they can reflect on themeslves*,* which improves their knowledge and helps polish their skills going forward.” (Int19P7)*.

Motivational framing further enhanced the developmental impact of feedback. Students reported feeling more empowered to take initiative when feedback recognized their progress and affirmed their capabilities and future readiness for autonomous practice. Although most preferred private settings for critical feedback, some valued public acknowledgment of strong performance, viewing it as a source of confidence, validation, and motivation to contribute more actively within the clinical setting:

*“If I did something good*,* I want to hear that I did something good in front of the team. It’s something to be proud of.” (Int1S1)*.

Collectively, these findings highlight the role of feedback as a developmental catalyst, supporting insight, strengthening reflective capacity, and preparing trainees for increasing levels of autonomy within pharmacy practice.

### Structural and relational tensions that constrain meaningful feedback

Although participants recognized feedback as a developmental rather than correctional process, they highlighted several structural and relational tensions that constrained its effectiveness. Time pressures, competing clinical and operational demands, and preceptors’ varying levels of comfort and skill in delivering feedback were perceived as significant barriers. Students also reported that power imbalances and limited psychological safety within some practice environments negatively influenced how feedback was received, at times leading them to disengage or dismiss preceptor input. Within the relational dynamics of feedback, some students described supervisors who were less open to dialogue about instructional preferences. Such responses were perceived as limiting open dialogue, contributing to relational strain and reducing students’ willingness to express their needs. As one student explained:

*“Some preceptors take it personally. Sometimes you’re scared they won’t accept feedback from you*,* and that makes you hesitant to speak up.”* (Int5S5).

The quality of language and tone used by preceptors was particularly influential. When feedback was phrased negatively or delivered in a manner perceived as accusatory, preceptors described students becoming defensive, which would consequently create a relational strain and reduce their willingness to act on the feedback. As one preceptor noted:

*“Negative feedback would be seen as blame rather than highlighting points for improvement… the language matters a lot when you provide feedback.”* (Int19P7).

Tensions also emerged from learner-related factors. Preceptors described instances where students demonstrated resistance to feedback, limited accountability, or what they perceived as a lack of willingness to change. Such behaviors diminished preceptors’ motivation to coach and disrupted the relational foundation necessary for meaningful feedback:

*“When you show them how to do it and they still don’t do it… a slightly careless attitude would be discouraging to ongoing mentorship.”* (Int19P7).

Structural and operational realities further compounded these relational tensions. Participants acknowledged that heavy workloads and service demands often took precedence over teaching, resulting in irregular or rushed feedback. When these pressures were persistent, students expressed frustration with the limited depth and consistency of the feedback they received.

Both groups emphasized the need for protected time dedicated to structured, high-quality feedback to support learning and ensure accountability from both preceptors and trainees. Students repeatedly emphasized that on-the-spot feedback allowed them to adjust their performance in real time and demonstrate visible improvement. Preceptors echoed this view, noting that consistent formative feedback cultivated autonomy and confidence, enabling trainees to take on increasing levels of responsibility.

### Building a shared feedback culture through clearer expectations, co-regulation, and enhanced preceptor support

Participants emphasized that meaningful feedback does not occur in isolation but depends on clear structures, shared expectations, and mutual responsibility within the learning environment. Both students and preceptors described goal-setting at the start of a rotation as essential for aligning expectations, creating accountability, and guiding feedback throughout the placement. When learning objectives were explicit and collaboratively negotiated, students reported feeling more equipped to interpret, apply, and benefit from feedback, viewing it as part of their broader professional development. Embedding this co-regulation within the developmental process consequently allows for meaningful feedback dialogue between preceptor and student. One preceptor articulated how establishing clear expectations at the start of the rotation promoted accountability and facilitated objective goal setting:

*“It’s important to explain to the student what is expected from the beginning*,* so they understand the tasks they should gradually take on across the four weeks. That’s why initial communication is really important to establish*,* let’s say*,* an agreement between the preceptor and the student.”* (Int13P1).

Despite this, students consistently noted significant variability in the quality, depth, and frequency of feedback across preceptors and practice sites. While some preceptors offered rich, specific, and structured feedback, others provided comments that were brief, inconsistent, or nonspecific. Participants expressed concern that such disparity not only created inequitable learning experiences but also limited the developmental impact of feedback. Both groups highlighted the need for enhanced preceptor training and calibration to support consistent, fair, and educationally meaningful feedback delivery:

*“In my experience some preceptors provided rich feedback*,* some preceptors did not. So it was not consistent. I think it’s important for them to be trained on how to properly provide feedback for students that would help the students improve themselves.”* (Int4S4).

Preceptors acknowledged these concerns, noting that existing training was infrequent or outdated. They expressed interest in more regular professional development and in platforms to share experiences, observe peers, and learn from one another. Many viewed peer-to-peer learning as an untapped resource with potential to enhance feedback practices and strengthen preceptor identity as educators:

*“If we spend time with each other doing activities together… we can learn from each other. Peer feedback would be nice for the future.” (Int13P1)*.

Participants also highlighted gaps in institutional feedback systems, particularly the absence of mechanisms for sharing student evaluations with preceptors. Preceptors felt that receiving structured feedback on their own performance was critical for reflecting on their teaching, improving their feedback delivery, and fostering transparency and accountability. They described closing this loop as essential for creating a culture in which both learners and educators are supported in their development, as noted by one preceptor:

*“Providing programs in a blended setting as workshops as well as seminars…would help us to identify our needs…and be constantly updated because I feel this is an area under preceptorship that is crucial.”* (Int23P10).

Collectively, these insights demonstrate that effective feedback extends beyond individual skills and requires building a broader feedback culture grounded in shared expectations, bidirectional dialogue, and institutional structures that support preceptor development and educational consistency.

## Discussion

This study explored the relational dynamics shaping how students and preceptors experience and internalize feedback within experiential learning, highlighting that feedback is a complex psychosocial process rather than a simple transmission of information. Participants consistently described feedback as fundamentally relational, shaped by trust, psychological safety, and mutual understanding – factors that strongly influenced how feedback was delivered, interpreted, and acted upon. They also emphasized that constructive feedback plays a central role in fostering trainee insight, reflection, and metacognitive development, thereby supporting ongoing professional growth. However, when structural and relational tensions emerged – such as limited time for feedback discussions, power differentials between preceptors and trainees, or varying levels of preceptor skills in giving feedback – students struggled to meaningfully internalize and apply feedback. These findings reinforce that feedback extends far beyond verbal comments; it is a nuanced psychosocial experience shaped by relational dynamics where preceptor identity and the quality of the preceptory relationship influence how feedback is experienced and internalized by learners.

Participants in this study described feedback as a fundamentally relational process shaped by trust, psychological safety, and the perceived credibility of the preceptor; factors that powerfully influenced how feedback was delivered, interpreted, and ultimately internalized. Both preceptors and students emphasized that preceptors who “led by example,” demonstrated professional integrity, and provided clear, constructive guidance were more successful in engaging learners and fostering receptivity to feedback. These insights align with the work of Watling and Ginsburg, who argue that credibility is central to effective feedback and is inherently embedded within the relational dynamics of supervision [19, 43, 44]. Harrison and colleagues similarly note that learners often disregard feedback they perceive as lacking credibility [13], affirming the importance of relational trust in shaping feedback uptake. Telio and colleagues further articulate that the educational alliance – rooted in mutual respect, shared goals, and a psychologically safe environment – is a critical foundation for meaningful feedback interactions [14]. Power dynamics are inherent within the preceptor–learner relationship; however, when perceived as imbalanced, they may contribute to psychological discomfort, which in turn can lead trainees to disengage from or dismiss feedback, as reflected in our participants’ accounts [45]. The experiences of participants in our study suggest that preceptorship extends beyond organizational oversight and the delivery of feedback. Rather, it is embedded within professional leadership, where the preceptor’s role modeling influences how feedback is enacted and received by learners.

In our study, participants described credibility as emerging through two interconnected dimensions: the preceptor’s role-modelling and mentorship behaviors, and the relational climate of psychological safety. These dimensions are essential, as trainees must feel supported and secure enough to reveal uncertainties, demonstrate vulnerability, and engage in honest reflection with preceptors who can mentor and coach them through guidance and empowerement [19, 46, 47]. When these conditions were present, students were more willing to participate actively in the feedback process, enhancing the developmental impact of feedback. Our findings extend the work of Schut and colleagues [17] on teacher-learner relationships in assessment by demonstrating that, within experiential pharmacy education, preceptors’ positional authority and relational investment shaped how learners interpreted and enacted feedback, ultimately influencing whether it was internalized as judgement or experienced as coaching.

Participants also emphasized that feedback served as a catalyst for professional growth, particularly when it was timely, specific, and learner-centered. Such feedback supported the development of metacognitive skills, enabling trainees to become more aware of their strengths and limitations while fostering reflective habits essential for their evolving professional identity. These findings align with the broader medical education literature, which consistently demonstrates that feedback is most effective when anchored in actionable guidance, delivered close to the performance event, and situated within mutually agreed-upon learning goals [30, 48]. This approach empowers trainees to assume greater ownership of their learning and remain actively engaged in feedback processes [46, 49, 50].

Our findings highlight the centrality of learner-centered feedback in promoting insight and metacognitive development [9] Prior research has noted that feedback conversations in clinical education often remain preceptor-dominated, with limited emphasis on the learner’s role in meaning-making and self-assessment, contributing to gaps in trainees’ insight into their own performance [9, 51]. In contrast, when feedback was framed constructively and oriented toward the trainee’s developmental needs, students in this study were more likely to internalize the guidance, engage in reflection, and develop the insight required for lifelong learning and adaptive expertise [52]. Preceptors described intentionally shaping their feedback to scaffold reflection – an approach that reflects an implicit shift from evaluator to coach, aligning with contemporary models of feedback that prioritize co-regulation, dialogic exchange, and the cultivation of reflective practitioners [46].

Structural and relational challenges also emerged as significant obstacles to effective feedback delivery. Participants described how time pressures, competing workload demands, and preceptors’ varying levels of confidence in giving feedback shaped the quality of learning conversations and influenced how students engaged with and internalized feedback. The language and tone used by preceptors were critical determinants of how feedback was received; feedback perceived as harsh, accusatory, or poorly phrased often triggered defensiveness, limiting students’ ability to act on it. These findings echo Brett and Atwater’s observations that learners who experience stress or threat during feedback are more likely to discount or devalue its content [53], and align with Urquhart and colleagues’ work illustrating that learners can experience feedback as something done to them rather than with them [2].

Our study reinforces that the relational climate surrounding feedback shapes whether feedback fosters growth or contributes to disengagement [23, 54]. While the presence of negatively perceived feedback is unavoidable in clinical training, the findings from our study support earlier literature advocating for learner-centered feedback that is task-focused, actionable, and framed to support improvement rather than judgement [8, 9, 19]. Importantly, participants highlighted that the effectiveness of feedback is influenced not only by preceptor behavior but also by learner attitudes; students who demonstrated defensiveness or limited insight also contributed to relational strain. Addressing this dynamic requires attention to both educator practices and learner cognitive and reflective capacities [9], positioning feedback as a shared relational responsibility rather than a unidirectional instructional act.

Our study highlights the importance of recognizing feedback as a bidirectional process in which both preceptor and learner actively contribute. When feedback is experienced as a bidirectional relationship rather than a transactional exchange, learners are more likely to experience psychological safety, engage meaningfully in the process, and strengthen their reflective capabilities [19, 55] A paradigm shift toward conceptualizing feedback as a collaborative, relational process – where preceptors and learners learn with and from each other – is essential to fostering an environment that nurtures growth, supports reflective practice, and enhances the quality of workplace-based learning.

This study has several limitations that should be considered when interpreting the findings. As with all qualitative research, the perspectives captured reflect the experiences of participants within a specific institutional and cultural context, which may not be representative of other pharmacy programs, healthcare settings, or health disciplines. Although purposive sampling ensured variation in participant backgrounds, those who elected to participate may have held stronger or more formed opinions about feedback processes, introducing the possibility of self-selection bias. Finally, this study explored participants’ self-reported experiences rather than directly observing feedback interactions, limiting insight into how feedback is enacted in real time and how relational dynamics unfold within authentic workplace encounters.

## Conclusion

By exploring the lived experiences of students and preceptors within workplace-based learning, this study reframes feedback as a bidirectional relational process grounded in credibility, psychological safety, and shared goal-setting. When constructive feedback is timely, specific, and delivered with clarity and respect, it fosters the development of metacognitive skills in students that are essential for lifelong learning and professional growth. As the scope of pharmacy practice continues to expand, feedback that is collaborative and developmentally oriented is best positioned to nurture trainee autonomy and support their progression toward the increasing responsibilities of contemporary pharmacy practice.

## Supplementary Information

Supplementary Material 1.

Supplementary Material 2.

## Data Availability

The anonymized interview transcripts that support the findings of this study are not publicly available due to ethical and institutional restrictions. However, the data may be made available upon reasonable request, subject to approval by the Hamad Medical Corporation Medical Research Center (MRC-01-23-426) and the Qatar University Institutional Review Board (1987201-1).
