# Supplementary material for: Feedback as a two-way process: how relational dynamics shape learning in workplace-based training
Source: BMC Med Educ. 2026 May 18;26:1116. doi: 10.1186/s12909-026-09462-7 (PMC13352886; doi:10.1186/s12909-026-09462-7)
Supplement: Supplementary file 1 — Supplementary Material 1. [file 12909_2026_9462_MOESM1_ESM.docx]

**Appendix A: Interview guide that informed the student and preceptor interviews, with a guide developed for each cohort accordingly.**
